# Effect of thoracic radiotherapy dose on the prognosis of advanced lung adenocarcinoma harboring EGFR mutations

**DOI:** 10.1186/s12885-022-10095-4

**Published:** 2022-09-24

**Authors:** Hongyue Qin, Jiaojiao Ke, Shuai Dong, Huani Li, Kunli Zhu, Shuai Fu, Qian Shao, Zhenxiang Li

**Affiliations:** 1 Department of Radiation Oncology, Shandong Cancer Hospital and Institute, Shandong First Medical University and Shandong Academy of Medical Sciences, Shandong Province 250117 Jinan, China; 2 Shandong First Medical University and Shandong Academy of Medical Sciences, Jinan, 250117 Shandong Province China; 3 Weihai Central Hospital, Weihai, Shandong Province China; 4 Department of Medical Imaging, Shandong Cancer Hospital and Institute, Shandong First Medical University and Shandong Academy of Medical Sciences, Jinan, 250117 Shandong Province China; 5 Heze Municipal Hospital, Heze, Shandong Province China; 6 Department of Internal Medicine-Oncology, Shandong Cancer Hospital and Institute, Shandong First Medical University and Shandong Academy of Medical Sciences, Jinan, 250117 Shandong Province China

**Keywords:** Radiotherapy dose, Epidermal growth factor receptor mutation, Advanced lung adenocarcinoma, Radiation pneumonia

## Abstract

**Background:**

The aim of this study was to investigate the effects of different thoracic radiotherapy doses on OS and incidence of radiation pneumonia which may provide some basis for optimizing the comprehensive treatment scheme of these patients with advanced EGFR mutant lung adenocarcinoma.

**Methods:**

Data from 111 patients with EGFR-mutant lung adenocarcinoma who received thoracic radiotherapy were included in this retrospective study. Overall survival (OS) was the primary endpoints of the study. Kaplan–Meier method was used for the comparison of OS. The Cox proportional-hazard model was used for the multivariate and univariate analyses to determine the prognostic factors related to the disease.

**Results:**

The mOS rates of the patients, who received radiotherapy dose scheme of less than 50 Gy, 50–60 Gy (including 50 Gy), and 60 Gy or more were 29.1 months, 34.4 months, and 51.0 months, respectively (log-rank *P* = 0.011). Although trend suggested a higher levels of pneumonia cases with increasing radiation doses, these lack statistical significance (χ^2^ = 1.331; *P* = 0.514). The multivariate analysis showed that the thoracic radiotherapy dose schemes were independently associated with the improved OS of patients (adjusted hazard ratio [HR], 0.606; 95% CI, 0.382 to 0.961; *P* = 0.033).

**Conclusions:**

For the patients with advanced *EGFR*-mutant lung adenocarcinoma, the radical thoracic radiotherapy dose scheme (≥ 60 Gy) could significantly prolong the OS of patients during the whole course management.

## Background

Oncogenic mutations in epidermal growth factor receptor (EGFR) are used as predictive biomarkers for using EGFR TKI as a treatment strategy for non-small cell lung cancer (NSCLC) [1, 2]. Therapeutic strategies, targeting EGFR, have greatly improved the progression-free survival (PFS) and overall survival (OS) of the patients with NSCLC, especially in the non-smoking females with lung adenocarcinoma and 80% *EGFR* mutation rate [3–7]. However, almost all the NSCLC patients develop drug resistance and suffer from the disease progression after experiencing about 8–12 months of PFS [8]. The common drug resistance mechanisms for EGFR TKIs include mutations in *T790M, KRAS, PIK3CA*, and *BRAF* genes, c-MET amplification, phenotype transformation (small cell lung cancer phenotype, stem cell phenotype), intra-tumoral genetic heterogeneity, etc. [9, 10].

EGFR TKIs in combination with other treatment schemes have emerged as a promising treatment strategy to overcome the drug’s resistance to EGFR TKIs treatment and further improve the prognosis for the patients [11–13]. NEJ009 study showed that gefitinib in combination with chemotherapy demonstrated a better objective response rate (ORR) and PFS of the patients with advanced *EGFR* mutant NSCLCs as compared to those treated with gefitinib alone. The median OS of the patients in the combination group was also significantly longer than those in the gefitinib group [11]. NEJ023 study showed that the combination therapy of bevacizumab and erlotinib improved PFS as compared to the erlotinib therapy alone in the patients with EGFR-positive NSCLC [12]. Both the clinical studies demonstrated that the EGFR TKIs in combination with other systemic treatment drugs could improve the patients’ survival. However, this type of combination treatment increased toxicities, which might be only suitable for patients with good performance status.

Radiotherapy as a local treatment regimen plays an important role in cancer management during the whole course of NSCLCs. Our previous study showed that the addition of thoracic radiotherapy in the advanced NSCLC patients with *EGFR* mutation could significantly improve their OS but choosing the optimal thoracic radiotherapy dose was unclear. For advanced NSCLC patients, radiotherapy is often used as a palliative treatment to improve the patients’ symptoms. Most of the patients with advanced lung adenocarcinoma received a lower dose of thoracic radiotherapy as compared to that of radical radiotherapy. The effects of high thoracic radiotherapy doses on improving the prognosis are still unclear. In addition, the patients, receiving thoracic radiotherapy, are at risk of developing radiation pneumonia [14]. Although an increase in thoracic radiotherapy dose could increase the local control of tumors, it also increased the risk of radiation pneumonia and affected the quality of patients’ lives [15].

Therefore, in this study, the patients with advanced *EGFR* mutant lung adenocarcinoma, who received thoracic radiotherapy, were retrospectively recruited. The effects of different thoracic radiotherapy doses on the OS of patients and incidence of radiation pneumonia were analyzed, which might provide a basis for optimizing a comprehensive treatment scheme for the patients.

## Methods

### Study population and data collection

One hundred eleven patients with *EGFR* mutant advanced NSCLC, who were treated in Shandong cancer hospital, were screened from September 2008 to April 2019. The inclusion criteria were as follows: (1) the patients were pathologically diagnosed with primary lung adenocarcinoma; (2) the patients had mutations in exons 18, 19, 20, or 21 of their *EGFR* gene; (3) the disease stage was determined using computed tomography (CT), magnetic resonance imaging (MRI), positron emission tomography CT, or invasive examination (aspiration cytology); (4) the detailed basic information and treatment information of were provided; (5) the patients received EGFR-TKIs treatment (gefitinib, erlotinib, osimertinib) and thoracic radiotherapy; and (6) the patients with second primary tumor were excluded. The basic information of the patients was collected as follows: age, sex, smoking history, tumor node metastasis classification (TNM), *EGFR* mutation type, diagnosis date, recurrence date, and follow-up and treatment information, which included radiotherapy dose, start time of the targeted treatment, and thoracic CT after radiotherapy. The staging of NSCLC was based on the TNM Staging Manual of the 8th edition of the American Joint Commission on Cancer (AJCC). The EGFR mutations were tested according to the professional gene testing methods. The diagnosis date was based on the pathological diagnosis time. The treatment plan was obtained by investigating the patient’s medical records. The follow-up of the patients was conducted using telephone interviews. The study was approved by the ethics committee of Shandong cancer hospital. The study was conducted following the Helsinki declaration.

### Treatment and follow-up

All the patients received EGFR TKIs (gefitinib, osimertinib, and erlotinib) as the first-line or second-line treatment. The patients also received other systemic treatments, including platinum, pemetrexed, gemcitabine, vinorelbine, docetaxel, albumin paclitaxel, and bevacizumab. In addition, the patient received thoracic or other organs (brain, bone, adrenal gland, liver, or spleen) radiotherapies. The radiotherapy for the primary lung tumors and thoracic metastatic lymph nodes is classified as thoracic radiotherapy. The dose for thoracic radiotherapy ranged from 2 to 4 Gy per dose and the total dosage scheme ranged from 30 to 70 Gy. According to the bioequivalent dose formula, the hypo-fractionated dose (3 and 4 Gy per fraction) was converted into a conventionally fractionated dose (2 Gy per fraction) and the total dose of thoracic radiotherapy was calculated. Radiation pneumonia was comprehensively diagnosed according to the patient’s radiotherapy history, actual symptoms, and pulmonary imaging results. Imaging diagnosis was based on the standards of the radiation therapy oncology group (RTOG) and the clinical symptoms depending on the treatment contents of previous medical records. The patients, who underwent thoracic CT examination for 1–6 months after radiotherapy for observation, were selected. The grade of radiation pneumonia was jointly evaluated by radiologists and imaging experts.

Regular follow-up and examinations after the treatment course were recommended for all the patients. In the first two years after treatment, thoracic CT was performed every 3 months and the patients were revisited. In the following 3 years, thoracic CT was performed every 6 months and the patients were revisited. Then, the thoracic CT was performed once a year and the patients were revisited. If the patient was unwell, the thoracic CT was performed in time. The follow-up information was obtained by telephone interviews or consulting the patient's medical records. The OS referred to the time from disease diagnosis to death.

### Statistical analyses

All the patients’ characteristics data were descriptively analyzed and compared. All the statistical analyses were conducted using IBM SPSS 25.0. Chi-square (χ^2^) test was performed to compare the categorical variables. Kaplan–Meier method was used for the comparison of OS. The Cox proportional-hazard model was used for the multivariate and univariate analyses to determine the prognostic factors related to the disease, and the hazard ratios were reported as relative risks with their corresponding 95% confidence intervals (CIs). *P* < 0.05 was considered statistically significant.

## Results

### Characteristics & treatments of the patients

This study enrolled a total of 111 patients, who received thoracic radiotherapy from September 2008 to April 2019. The majority of the patients were females (71 patients, accounting for 63.96%) and non-smokers (90 patients, accounting for 81.08%). Among the 111 patients, 80 patients (accounting for 72.07%) died and 31 patients (accounting for 27.93%) survived. Their median age was 53 (ranged: 32–82). There were 32 people (accounting for 28.83%) over the age of 60 (including 60). For the *EGFR* mutation type analysis, mutations in exon 21 and 19 in all patients accounted for 45.05% and 44.14%, respectively. The sites of the patients targeted with radiotherapy mainly included thorax (111 patients, accounting for 100%), brain (60 patients, accounting for 54.05%), and bone (47 patients, accounting for 42.34%). There were 69 cases (accounting for 62.16%) of grade 1 radiation pneumonia, 10 cases (accounting for 9.01%) of grade 2 pneumonia, and 4 cases (accounting for 3.60%) of grade 3 pneumonia, respectively. Of the 111 patients, who received thoracic radiotherapy, 25 patients (accounting for 22.52%) received less than 50 Gy, 35 patients (accounting for 31.53%) received 50–60 Gy (including 50 Gy) and 51 patients (accounting for 45.95%) received 60 Gy or more. A total of 19 patients (accounting for 17.12%) were treated with targeted combined radiotherapy concurrently. The clinicopathological characteristics of the patients are summarized in Table 1.

**Table 1:** Characteristics of 111 advanced lung adenocarcinoma patients

Characteristic	Total (*N* = 111)	%
**Sex**		
Male	40	36.04%
Female	71	63.96%
**Age, n (%)**		
Median age, y (range)53		
≥ 60	32	28.83%
< 60	79	71.17%
**Tobacco smoking, n (%)**		
yes	21	18.92%
no	90	81.08%
**EGFR mutation, n (%)**		
19	49	44.14%
21	50	45.05%
other	12	10.81%
**Dose, Gy**		
< 50	25	22.52%
≥ 50, < 60	35	31.53%
≥ 60	51	45.95%
**Number of radiotherapy sites**		
1	28	25.23%
2	52	46.85%
3	24	21.62%
4	3	2.70%
5	3	2.70%
6	1	0.90%
**The Site of Radiotherapy**		
Thoracic	111	100.00%
Brain	60	54.05%
Bone	47	42.34%
**Radiation pneumonitis**		
G1	69	62.16%
G2	10	9.01%
G3	4	3.60%
**Radiotherapy targeting synchronization**		
yes	19	17.12%
no	92	82.88%

*Abbreviations: EGFR* Epidermal growth factor receptor, *TKIs* Tyrosine kinase inhibitors

### Survival status of the entire study cohort

**Fig. 1:**
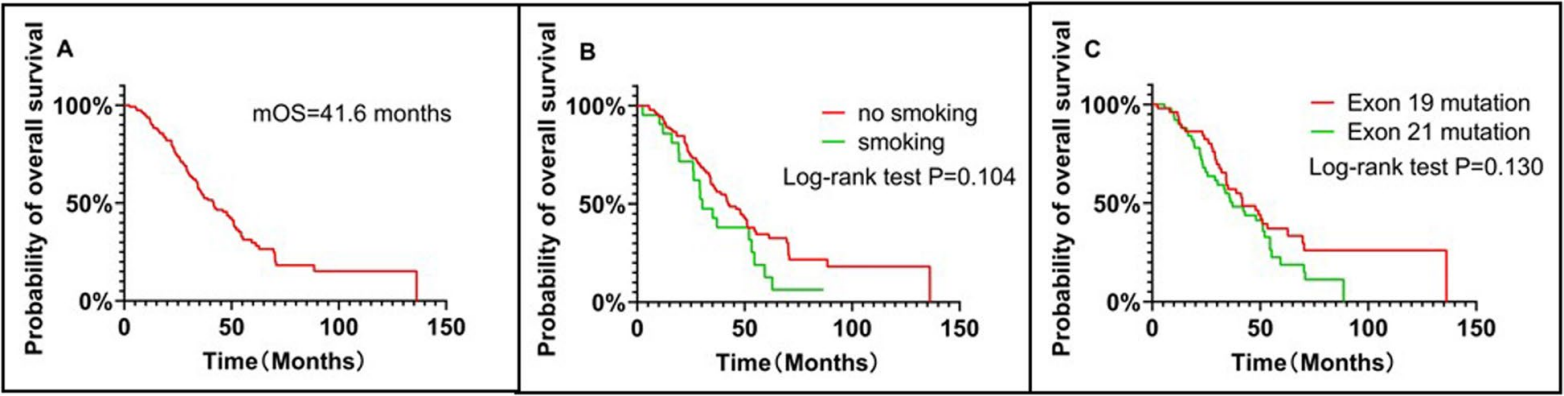
Overall survival (OS) of the entire cohort (**A**) and smoking status(**B**) and of patients stratified according to EGFR mutation status (**C**)

**Fig. 2:**
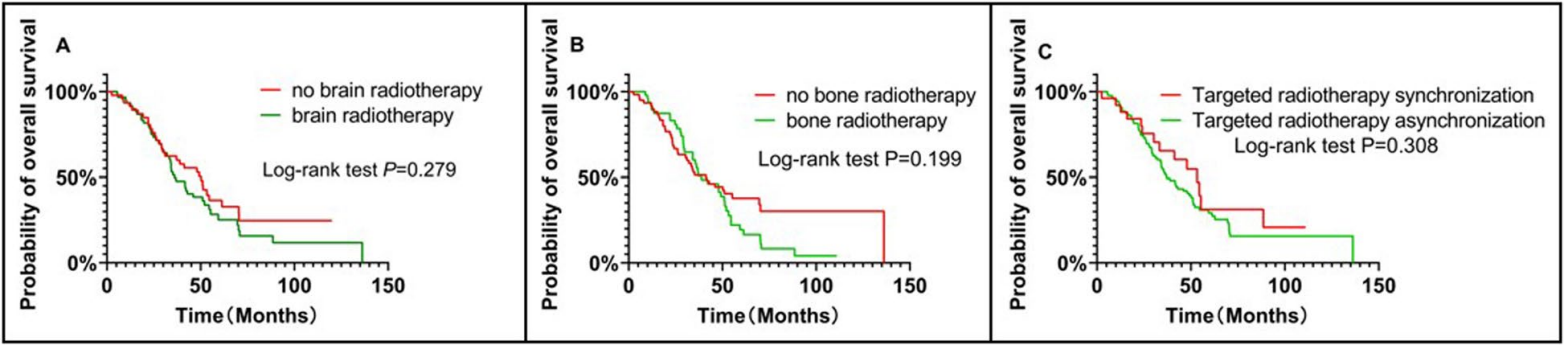
Effect of brain radiotherapy on overall survival (OS) in patients with brain metastasis (**A**). Effect of bone radiotherapy on overall survival (OS) in patients with bone metastasis (**B**). Effect of targeted combined radiotherapy on overall survival (OS) in the entire cohort (**C**)

The OS of the patients was defined as the duration from the beginning of therapy to death from any cause or the last follow-up. The patients, who were lost to follow-up or survived until the end of this study observation time were defined as censored. After the follow-up duration, there were 80 patients (accounting for 72.07%) in the entire cohort. The median overall survival (mOS) of the patients was 41.6 months (Fig. 1A). The rates of 1-, 3-, and 5-year OS rates among the entire cohort were 91.89%, 54.36%, and 28.25%, respectively. There were no significant differences in OS rates between the 90 non-smokers and 21 smokers (hazard ratio(HR) = 0.651; 95% CI 0.358 to 1.186; log-rank *P* = 0.104; Fig. 1B).The mOS rates of the patients with exons 19 and 21 mutations were 41.7 months and 37.1 months, respectively (HR = 0.695; 95% CI 0.434 to 1.113; log-rank* P* = 0.130; Fig. 1C). There was no significant difference in the OS rate of the 60 patients who received brain radiotherapy and those who did not receive brain radiotherapy (HR = 0.778; 95% CI 0.494 to 1.225; log-rank *P* = 0.279; Fig. 2A). Among the 79 patients with bone metastases, there was no significant difference in the OS rates between 47 patients who received bone radiotherapy and those who did not receive bone radiotherapy (HR = 0.751; 95% CI 0.479 to 1.175; log-rank *P* = 0.199; Fig. 2B). The difference in the OS of the 19 patients, receiving targeted immunotherapy combined with radiotherapy synchronously and those, receiving radiotherapy combined with targeted immunotherapy asynchronously was not statistically significant (HR = 0.743; 95% CI 0.439 to 1.259; log-rank *P* = 0.308; Fig. 2C).

### Effect of different thoracic radiotherapy doses on the patients’ OS and incidence of radiation pneumonia

**Fig. 3:**
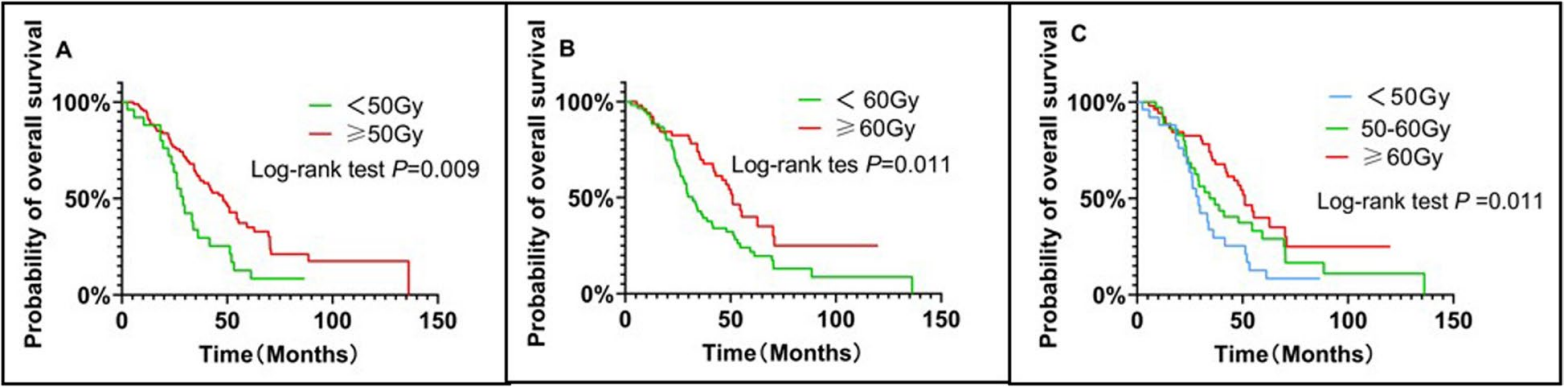
Comparison of OS between < 50 Gy and ≥ 50 Gy groups (**A**). Comparison of OS between < 60 Gy and ≥ 60 Gy groups (**B**). Overall survival (OS) of patients according to different thoracic radiotherapy doses (**C**)

The effects of different lung radiotherapy doses on the patients’ OS were analyzed. The patients were divided into three groups based on the different doses. This difference was statistically significant in the OS of the patients who received a radiotherapy dose scheme of less than 50 Gy, and those who received a radiotherapy dose scheme of more than 50 Gy (including 50 Gy)(HR = 0.457; 95% CI 0.253 to 0.826; log-rank *P* = 0.009; Fig. 3A). The difference in the OS of patients receiving less than 60 Gy and those who received more than 60 Gy (including 60 Gy) was statistically significant (HR = 0.628; 95% CI 0.399 to 0.987; log-rank *P* = 0.011; Fig. 3B). The mOS rates of the patients who received radiotherapy dose scheme of less than 50 Gy, 50–60 Gy (including 50 Gy), and 60 Gy or more were 29.1 months, 34.4 months, and 51.0 months, respectively (log-rank *P* = 0.011). The patients who received a radiotherapy dose scheme of 60 Gy or more had a longer mOS rate as compared to the other two groups (HR = 0.628; 95% CI 0.399 to 0.987; log-rank *P* = 0.011; Fig. 3C).

**Fig. 4:**
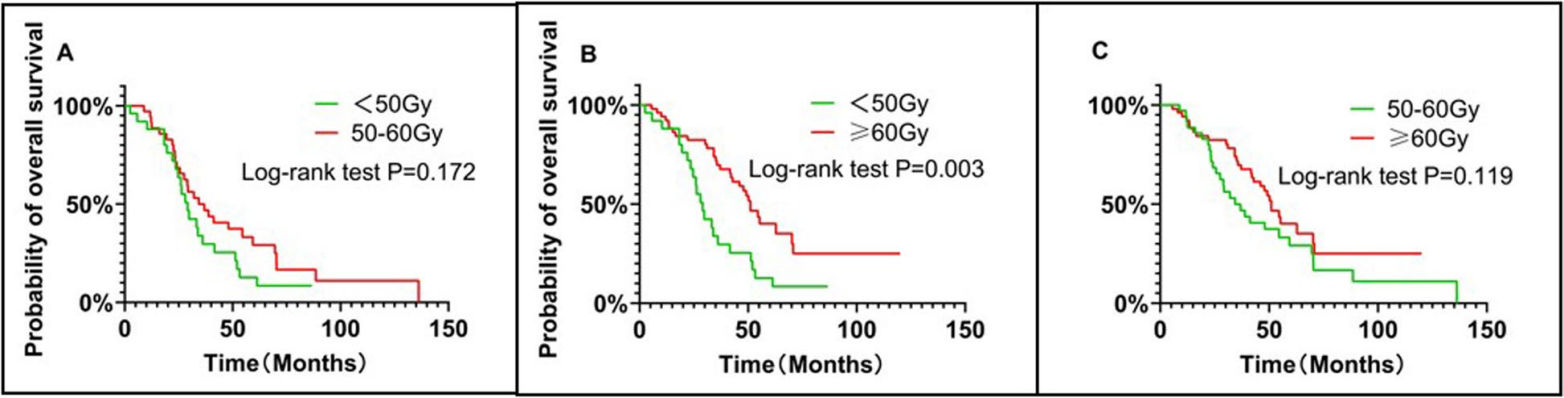
Comparison of OS between < 50 Gy and 50-60 Gy groups (**A**). Comparison of OS between < 50 Gy and ≥ 60 Gy groups (**B**). Comparison of OS between 50-60 Gy and ≥ 60 Gy groups (**C**)

The subgroup analysis revealed that this difference was not statistically significant in the OS of the patients who received a radiotherapy dose scheme of 50–60 Gy (including 50 Gy), and those who received a radiotherapy dose scheme of less than 50 Gy(mOS: 34.4 months *vs.* 29.1 months; HR = 0.684; 95% CI 0.383 to 1.223; log-rank *P* = 0.172; Fig. 4A). The mOS of patients who received a radiotherapy dose scheme of 60 Gy or more was longer than that of the patients who received a radiotherapy dose scheme of less than 50 Gy (mOS: 51.0 months *vs.* 29.1 months, HR = 0.444; 95% CI 0.236 to 0.835; *P* = 0.003; Fig. 4B). This difference inclined to statistically insignificant in the OS rates of 56 patients who received radiotherapy dose scheme of 60 Gy or more, as compared to those who received radiotherapy dose scheme of 50-60 Gy (including 50 Gy) (mOS: 51.0 months *vs.* 34.4 months, HR = 0.672; 95% CI 0.396 to 1.141; *P* = 0.119; Fig. 4C).

The results showed that the incidence of grade 1 and 2 radiation pneumonia was 21.52% in the patients receiving radiotherapy dose scheme of less than 50 Gy, 30.38% in the patients receiving radiotherapy dose scheme of 50 to 60 Gy (including 50 Gy), 48.10% in the patients receiving radiotherapy dose scheme of 60 Gy or more. There was no patients who developed grade 3 radiation pneumonia in the group of radiotherapy dose scheme of less than 50 Gy. Two patients in those receiving radiotherapy dose schemes of 50–60 Gy developed grade 3 radiation pneumonia. Two patients in those receiving radiotherapy dose schemes of 60 Gy or more developed grade 3 radiation pneumonia (χ^2^ = 1.331; *P* = 0.514; Table 2). Although the incidence of radiation pneumonia in the group of high doses was higher than that in other two groups, the statistical difference was not reached (χ^2^ = 1.331; *P* = 0.514; Table 2). The severity of radiation pneumonia was not related to the synchronization of targeted immunotherapy and radiotherapy (χ^2^ = 0.152; *P* = 0.697; Table 3).

**Table 2:** Characteristics of patients in the three groups and with the X2 test for categorical variables

Characteristic		< 50 Gy	≥ 50 Gy, < 60 Gy	≥ 60 Gy	*P*
Sex					
Male	40 (36.04%)	13	11	16	0.168
Female	71 (63.96%)	12	24	35	
Age, n (%)					
Median age, y (range)53					
≥ 60	32 (28.83%)	10	8	14	0.337
< 60	79 (71.17%)	15	27	37	
Tobacco smoking, n (%)					
yes	21 (18.92%)	9	5	7	0.032
no	90 (81.08%)	15	30	45	
EGFR mutation, n (%)					
19	49 (44.14%)	5	19	25	0.065
21	50 (45.05%)	15	14	21	
Number of radiotherapy sites					
1、2、3	104(93.70%)	23	31	50	0.192
4、5、6	7 (6.30%)	2	4	1	
Radiation pneumonitis					
1、2	79 (71.18%)	17	24	38	0.514
3	4 (3.60%)	0	2	2	
Radiotherapy targeting synchronization					
yes	19 (17.12%)	4	9	6	0.237
no	92 (82.88%)	21	26	45

**Table 3:** Characteristics of patients in the two groups and with the X2 test for categorical variables

	**Radiotherapy targeting synchronization**		
	**Yes**	**No**	***P***
**grade1 or 2**	19	90	0.697
**grade 3**	1	3	

### Univariate and multivariate analysis of independent predictors of overall survival

In order to exclude the influence of other clinicopathological factors on the OS of patients, χ^2^-test was used to analyze the differences in clinicopathological factors among the different dose groups. The non-smoking patients were more common among those, who received ≥ 60 Gy (16 patients in < 50 Gy group; 31 patients in ≥ 50 Gy group; 49 patients in ≥ 60 Gy group;* P* = 0.032;). There were no differences among the three groups for age, sex, *EGFR* mutation type, radiation pneumonia, treatment sequence of targeted immunotherapy and radiotherapy, or number of radiotherapy sites (Table 2).

The univariate and multivariate analyses of OS were performed using the COX regression model. The factors considered for these analyses included age, sex, smoking status, lung radiotherapy dose schemes, type of mutations in *EGFR* gene, sites of radiotherapy, number of radiotherapy sites, and treatment sequence of targeted immunotherapy and radiotherapy. The multivariate analysis showed that, after adjusting for the significant covariates, including thoracic radiotherapy doses, type of *EGFR* mutations, number of radiotherapy sites, sites of radiotherapy, and treatment sequence of targeted and radiotherapy, the thoracic radiotherapy dose schemes were independently associated with the improved OS of patients (adjusted hazard ratio [HR], 0.606; 95% CI, 0.382 to 0.961; *P* = 0.033; Table 4).

**Table 4:** Univariable and Multivariable analyses of covariable associated with OS

Variable	Univariate analysis		Multivariate analysis	
	HR (95% CI)	*P*	HR (95% CI)	*P*
Sex				
Male VS Female	0.875 (0.559 to 1.371)	0.563		
Age				
≥ 60 VS < 60	1.086 (0.678 to 1.739)	0.733		
Tobacco smoking				
yes VS no	0.651 (0.358 to 1.186)	0.104	0.785 (0.494 to 1.247)	0.305
EGFR mutation, n (%)				
19 VS 21	0.695 (0.434 to 1.113)	0.130	0.721 (0.421 to 1.233)	0.232
Number of radiotherapy sites				
1 VS 2–6	0.617 (0.377 to 1.010)	0.086	0.743 (0.413 to 1.335)	0.321
The Site of radiotherapy				
Brain yes VS no	0.778 (0.494 to 1.225)	0.279		
Bone yes VS no	0.751 (0.479 to 1.175)	0.199		
Dose, Gy		0.011		
< 50	0.457 (0.253 to 0.826)	0.009		
≥ 50, < 60	1.247 (0.788 to 1.974)	0.528		
≥ 60	0.628 (0.399 to 0.987)	0.011	0.606 (0.382 to 0.961)	0.033
Radiotherapy targeting synchronization				
yes VS no	0.743 (0.439 to 1.259)	0.308	0.758 (0.423 to 1.357)	0.351

*Abbreviations: OS* Overall survival

## Discussion

Radiotherapy is an important treatment strategy for the locally advanced NSCLCs [16]. Previous studies showed that the radiotherapy dose scheme of 60–66 Gy is an ideal radical radiation dose scheme, which can increase the OS of patients without increasing the incidence of radiation pneumonia for the locally advanced NSCLCs [17]. For the advanced NSCLC, the value of thoracic radiotherapy in the patients with *EGFR* mutant lung adenocarcinoma for the OS of patients has been previously investigated. The results showed that the patients, receiving thoracic radiotherapy had longer OS than those, who did not receive thoracic radiotherapy [18]. However, the effects of different radiotherapy dose schemes on the patients’ OS rates are needed to be further investigated. The radiotherapy dose scheme of less than 60 Gy is defined as palliative radiotherapy (PRT) and that of higher than 60 Gy (including 60 Gy) is defined as radical radiotherapy (RRT) [19–21]. In this study, the radiotherapy dose schemes were divided into three groups: 50 Gy (low PRT dose group), 50–60 Gy (including 50 Gy) high PRT dose group) and ≥ 60 Gy (RRT dose group). It showed that ≥ 60 Gy can obtain a longer OS compared to the other two groups. The results have a great clinical significance, indicating that the increased local control of thoracic lesions in the advanced *EGFR* mutant lung adenocarcinoma can improve OS, emphasizing the importance of local treatment strategy for primary lesions during the whole course of the disease.

Due to higher radiation doses, the greater the damage to normal lung tissues, the greater the probability of radiation pneumonia. This might ultimately affect the quality of survival and OS of the patients. In this study, there was a clear benefit in the prognosis of patients, receiving RRT dose, as compared to those, receiving PRT dose. There was no difference in the toxicity of PRT and RRT doses in the patients [22]. The most common adverse effect of radiotherapy was radiation pneumonia. The differences in the radiation pneumonia among the different groups were further analyzed. Although trend suggested a higher levels of pneumonia cases with increasing radiation doses, these lack statistical significance which may be caused by relatively small sample in our study. The thoracic radiotherapy dose for the patients, ranging from 2 to 4 Gy, for a total radiotherapy dose scheme of up to 60 Gy was generally well-tolerated and did not result in increased acute or subacute toxicity as compared to that of the lower dose schemes.

Individualized radiotherapy during the course of the disease showed a favorable safety profile and promising outcome, thereby serving as a therapeutic option for the patients with locally advanced or metastatic NSCLC [23]. Thoracic radiotherapy plays an increasingly important role in the stage IV lung adenocarcinomas (oligo-metastasis), harboring *EGFR* mutations [24]. Oligo-metastases mean limited or few metastases and are rarely defined further [25]. Generally, oligo-metastasis is defined as 1–5 metastatic lesions [26]. Among the patients with lung adenocarcinoma metastases, those with oligo-metastasis can achieve long-term survival from aggressive local management. The recent phase II randomized clinical trials showed that the ablative radiotherapy, including stereotactic ablative body radiotherapy (SABR) and hypo-fractionated radiotherapy, for the primary and metastatic sites improved the PFS and OS of the patients with oligometastatic NSCLC [27, 28]. For the patients with multiple sites metastases, the timely radiotherapy could significantly improve their performance status (PS), thereby affecting the prognosis [26]. The efficacy of different radiotherapy sites and optimal dose of radiotherapy need further investigations. A study about the failure pattern of EGFR TKI treatment showed the progression of cancer in over one-third of the patients at the original sites, which suggested that the combination of local treatment of the original sites could further improve the patients’ survival, as confirmed in our previous study [29, 30]. The incidence of radiation pneumonia was not related to the treatment sequence of targeted immunotherapy and radiotherapy. Through a regular follow-up, it was found that the tumor volume of *EGFR*-mutant lung adenocarcinoma decreased significantly during the application of targeted drugs for 2–3 months and then tended to become stable [31]. Therefore, it was suggested that the best time to strengthen the local control and reduce the incidence of radiation pneumonia might be the treatment with a thoracic radiotherapy scheme of more than 60 Gy after taking EGFR TKI drugs until the tumor volume is stabilized during follow-up.

This study had several limitations that could not be neglected. First, this was a retrospective single-institute-based study and the number of patients was relatively small. More patients with advanced NSCLC treated with radiotherapy should be enrolled and a multicenter collaborative prospective study should be conducted. Second, the effects of treatment schemes on prognosis were not analyzed after recurrence due to the unavailability of data. The results warrant a prospective validation in a larger cohort. A subset of the patients received systemic therapy or local therapy before TKI treatment, which might result in unknown subsequent effects on tumor response to TKI.

## Conclusions

This study showed that, for the patients with advanced *EGFR*-mutant lung adenocarcinoma, the radical thoracic radiotherapy dose scheme (≥ 60 Gy) could significantly prolong the OS of patients during the whole course management.

## Data Availability

The datasets generated for this study are available on request to the corresponding author.

## Abbreviations

EGFR: Epidermal growth factor receptor
TKI: Tyrosine kinase inhibitor. ORR, objective response rate
OS: Overall survival
PFS: Progression-free survival
TNM: Tumor node metastasis
mOS: Median overall survival
PRT: Palliative radiotherapy
RRT: Radical radiotherapy

## References

[1] Paez JG, Jänne PA, Lee JC, Tracy S, Greulich H, Gabriel S (2004). EGFR mutations in lung cancer: correlation with clinical response to gefitinib therapy. Science.

[2] Lynch TJ, Bell DW, Sordella R, Gurubhagavatula S, Okimoto RA, Brannigan BW (2004). Activating mutations in the epidermal growth factor receptor underlying responsiveness of non-small-cell lung cancer to gefitinib. N Engl J Med.

[3] Zhou C, Wu Y-L, Chen G, Feng J, Liu X-Q, Wang C (2011). Erlotinib versus chemotherapy as first-line treatment for patients with advanced EGFR mutation-positive non-small-cell lung cancer (OPTIMAL, CTONG-0802): a multicentre, open-label, randomised, phase 3 study. Lancet Oncol.

[4] Han J-Y, Park K, Kim S-W, Lee DH, Kim HY, Kim HT (2012). First-SIGNAL: first-line single-agent iressa versus gemcitabine and cisplatin trial in never-smokers with adenocarcinoma of the lung. J Clin Oncol.

[5] Mok TS, Wu Y-L, Thongprasert S, Yang C-H, Chu D-T, Saijo N (2009). Gefitinib or carboplatin-paclitaxel in pulmonary adenocarcinoma. N Engl J Med.

[6] Maemondo M, Inoue A, Kobayashi K, Sugawara S, Oizumi S, Isobe H (2010). Gefitinib or chemotherapy for non-small-cell lung cancer with mutated EGFR. N Engl J Med.

[7] Mitsudomi T, Morita S, Yatabe Y, Negoro S, Okamoto I, Tsurutani J (2010). Gefitinib versus cisplatin plus docetaxel in patients with non-small-cell lung cancer harbouring mutations of the epidermal growth factor receptor (WJTOG3405): an open label, randomised phase 3 trial. Lancet Oncol.

[8] Wu S-G, Shih J-Y (2018). Management of acquired resistance to EGFR TKI-targeted therapy in advanced non-small cell lung cancer. Mol Cancer.

[9] Tang Z-H, Lu J-J (2018). Osimertinib resistance in non-small cell lung cancer: mechanisms and therapeutic strategies. Cancer Lett.

[10] Rotow J, Bivona TG (2017). Understanding and targeting resistance mechanisms in NSCLC. Nat Rev Cancer.

[11] Hosomi Y, Morita S, Sugawara S, Kato T, Fukuhara T, Gemma A (2020). Gefitinib alone versus gefitinib plus chemotherapy for non-small-cell lung cancer with mutated epidermal growth factor receptor: NEJ009 study. J Clin Oncol.

[12] Saito H, Fukuhara T, Furuya N, Watanabe K, Sugawara S, Iwasawa S (2019). Erlotinib plus bevacizumab versus erlotinib alone in patients with EGFR-positive advanced non-squamous non-small-cell lung cancer (NEJ026): interim analysis of an open-label, randomised, multicentre, phase 3 trial. Lancet Oncol.

[13] Oizumi S, Sugawara S, Minato K, Harada T, Inoue A, Fujita Y (2018). Updated survival outcomes of NEJ005/TCOG0902: a randomised phase II study of concurrent versus sequential alternating gefitinib and chemotherapy in previously untreated non-small cell lung cancer with sensitive EGFR mutations. ESMO Open.

[14] Hanania AN, Mainwaring W, Ghebre YT, Hanania NA, Ludwig M (2019). Radiation-induced lung injury: assessment and management. Chest.

[15] Bledsoe TJ, Nath SK, Decker RH (2017). Radiation pneumonitis. Clin Chest Med.

[16] Xia B, Zhang S, Ma S (2017). Management of non-small cell lung cancer with mutation: the role of radiotherapy in the era of tyrosine kinase inhibitor therapy-opportunities and challenges. J Thorac Dis.

[17] Peters S, Felip E, Dafni U, Belka C, Guckenberger M, Irigoyen A (2019). Safety evaluation of nivolumab added concurrently to radiotherapy in a standard first line chemo-radiotherapy regimen in stage III non-small cell lung cancer-The ETOP NICOLAS trial. Lung Cancer.

[18] Zhang Y, Wang W, Xu X, Li Y, Zhang H, Li J (2021). Impact of radiotherapy pattern on the prognosis of stage iv lung adenocarcinomas harboring EGFR mutations. Cancer Manag Res.

[19] Lehman M, Bernard A, See A, King M, Michael M (2021). A randomized phase 3 trial of palliative radiation therapy versus concurrent chemotherapy and palliative radiation therapy in patients with good performance status, locally advanced, or metastatic non-small cell lung cancer with symptoms due to intrathoracic disease who are not suitable for radical chemo-radiation therapy: results of the trans-tasman radiation oncology group 11.03 trial. Pract Radiat Oncol.

[20] Toy E, Macbeth F, Coles B, Melville A, Eastwood A (2003). Palliative thoracic radiotherapy for non-small-cell lung cancer: a systematic review. Am J Clin Oncol.

[21] Saunders MI (2001). Programming of radiotherapy in the treatment of non-small-cell lung cancer–a way to advance care. Lancet Oncol.

[22] Katsui K, Ogata T, Watanabe K, Katayama N, Kuroda M, Kiura K (2020). Radiation pneumonitis after definitive concurrent chemoradiotherapy with cisplatin/docetaxel for non-small cell lung cancer: analysis of dose-volume parameters. Cancer Med.

[23] Noronha V, Patil VM, Joshi A, Menon N, Chougule A, Mahajan A (2020). Gefitinib versus gefitinib plus pemetrexed and carboplatin chemotherapy in -mutated lung cancer. J Clin Oncol.

[24] Zheng L, Wang Y, Xu Z, Yang Q, Zhu G, Liao X-Y (2019). Concurrent EGFR-TKI and thoracic radiotherapy as first-line treatment for stage IV Non-small cell lung cancer harboring EGFR active mutations. Oncologist.

[25] Stephens SJ, Moravan MJ, Salama JK (2018). Managing patients with oligometastatic non-small-cell lung cancer. J Oncol Pract.

[26] Zhou Y, Yu F, Zhao Y, Zeng Y, Yang X, Chu L (2020). A narrative review of evolving roles of radiotherapy in advanced non-small cell lung cancer: from palliative care to active player. Transl Lung Cancer Res.

[27] Timmerman RD, Kavanagh BD, Cho LC, Papiez L, Xing L (2007). Stereotactic body radiation therapy in multiple organ sites. J Clin Oncol.

[28] Suh Y-G, Cho J (2019). Local ablative radiotherapy for oligometastatic non-small cell lung cancer. Radiat Oncol J.

[29] Tang Y, Xia B, Xie R, Xu X, Zhang M, Wu K (2020). Timing in combination with radiotherapy and patterns of disease progression in non-small cell lung cancer treated with EGFR-TKI. Lung Cancer.

[30] Westover KD, Loo BW, Gerber DE, Iyengar P, Choy H, Diehn M (2015). Precision Hypofractionated radiation therapy in poor performing patients with non-small cell lung cancer: phase 1 dose escalation trial. Int J Radiat Oncol Biol Phys.

[31] Wang X, Zeng Z, Cai J, Xu P, Liang P, Luo Y (2021). Efficacy and acquired resistance for EGFR-TKI plus thoracic SBRT in patients with advanced EGFR-mutant non-small-cell lung cancer: a propensity-matched retrospective study. BMC Cancer.

